# COVID-19 Induced Stigmas of Imported Cold-Chain Food Among Chinese Consumers: Multi-Round Tracking Surveys

**DOI:** 10.3390/bs15040421

**Published:** 2025-03-25

**Authors:** Erpeng Wang

**Affiliations:** School of Economics and Management, Nanjing Tech University, Nanjing 211816, China; youxuels@163.com

**Keywords:** COVID-19, food stigma, risk perception, willingness to pay, imported cold-chain food

## Abstract

The COVID-19 pandemic has significantly disrupted the global food supply chain, concurrently eroding consumer trust in imported food products. We conducted multi-round tracking surveys of Chinese consumers nationwide in December 2020 and January, March, April, May, July, and October 2021 to examine the stigmas induced by COVID-19 on imported cold-chain food. Results revealed that COVID-19 has induced a long-lasting stigma of imported cold-chain food among Chinese consumers. The mean willingness to pay for beef steak from the U.S., Australia, and Brazil decreased by about 4 yuan, 3 yuan, and 3 yuan, respectively, compared to that for the corresponding product before the pandemic. The results also showed that respondents’ risk perception of imported cold-chain food decreased slightly in the short term and then stayed at a high level. Elderly consumers and those with children were more likely to perceive a high risk of imported cold-chain food associated with COVID-19. Our results indicated that the stigmas of imported cold-chain food caused by COVID-19 persistently impacted consumer food behavior. Identifying ways to recover consumer trust in imported food is essential to boost consumer demand with the recovery of the global food supply chain.

## 1. Introduction

Starting in 2020, the COVID-19 pandemic has hit hard on the world economy and global health (Nakat & Bou-Mitri, 2021). Although it is clear that the COVID-19 pandemic has disrupted the international food supply chain (Lüth et al., 2019), our knowledge about COVID-19-induced stigmas of imported cold-chain food among Chinese consumers, especially the stigmas in the long term, remains limited. Some research indicates that media coverage about associations between food and COVID-19 could lead to a persistent stigma of food (McFadden et al., 2021). Consumer food consumption behaviors will stick to the new normal and set a new trajectory for the future of the food industry (Chenarides et al., 2021). Meanwhile, others speculate that once we emerge from the aftermath of COVID-19, consumer food behaviors will return to the normal of pre-COVID-19. The conclusion on the impact of COVID-19 on food consumption, particularly on import food consumption, is still unclear.

Taking cold-chain beef as a case study, we conducted multi-round tracking surveys of Chinese consumers nationwide in December 2020 and January, March, April, May, July, and October 2021 to examine the stigmas induced by COVID-19 on imported cold-chain food. Specifically, the aim of this study is three-fold: (a) examine how testing positive for COVID-19 in cold-chain products affects consumers’ risk perception of imported beef steak, (b) identify its effect on consumers’ preferences for imported cold-chain food, and (c) determine the short- and long-term effects of COVID-19 on consumers’ preference changes. Although there is a large body of literature on the effect of COVID-19 on consumer food consumption behavior, we contribute to the literature from two distinct perspectives.

First, we are among the first to use multi-round tracking surveys to examine the short- and long-term effects of COVID-19 on consumer food consumption behavior. The exceptions are McFadden et al. (2021) and Tonsor et al. (2021). McFadden et al. (2021) explored U.S. consumer concerns about exposure to COVID-19 while shopping and consuming food in May, June, and July 2020. Tonsor et al. (2021) measured the impact of COVID-19 on U.S. meat consumers’ behaviors with a monthly tracking survey from February to July 2020. However, these tracking surveys only covered a period of the COVID-19 pandemic. Few studies have used seven-round surveys (December 2020 and January, March, April, May, July, and October 2021) to track consumer risk perceptions of imported cold-chain food and estimate consumer preferences for imported food over time. In addition, the time of these surveys is unique, covering a wide range of periods associated with different COVID-19 control statuses worldwide. For instance, our first survey was conducted in December 2020, when most countries still imposed restrictions on controlling COVID-19, while vaccines started getting approval (e.g., the U.S. FDA granted Pfizer-BioNTech and Moderna the first emergency use authorization (EUA) for an mRNA vaccine in December 2020). The other survey covers periods when countries worldwide were experiencing different COVID-19 pandemic statuses, vaccination requirements, and policy implementations. For instance, in the last week of 2020 (29 December to 5 January), the U.S. reported the first case of a new virus strain. The total death toll passed 4000 in the second week of 2021 (6 January 2021). In March 2021, the global COVID-19 infection rate increased sharply^1^. However, in April 2021, several U.S. states relaxed mask mandates, implementing a less strict COVID-19 control policy^2^. In May 2021, international travel resumed for vaccinated people in several countries. More vaccines received approval from the U.S. FDA and the World Health Organization (WHO), and an increasing population received the COVID-19 vaccine. By October 2021, many countries started abandoning zero COVID-19 strategies and relied more on the vaccine for COVID-19 control^3^. Using a rich amount of data from the seven-round surveys would provide valuable information on consumer risk perception and preference changes regarding imported food.

We further contribute to the literature by examining Chinese consumer preference for imported meat during the COVID-19 pandemic at different stages. Although there is some research on Chinese consumer preference for imported meat (Lin et al., 2022; Ortega et al., 2016), most of them were conducted in the pre-COVID-19 period. Because of COVID-19’s broad and substantial personal and social influence, it may not only act as a short-term shock to people’s behavior but also cause long-term persistent changes. Therefore, examining how COVID-19 affects consumer preferences for imported products in the short and long term is critical. This is particularly true for the Chinese market, the world’s largest agricultural importer. This research provides crucial information on developing strategies that resume consumers’ trust and demand for imported agricultural products when viruses such as COVID-19 disrupt the global food supply chain.

### 1.1. Background

Since multiple cold-chain products, like beef, pork, and poultry, have tested positive for SARS-CoV-2, some studies have suggested that there are critical links between the pandemic and the food system (Dyal, 2020). It has also caused an unprecedented global health crisis, creating potential risks to food security (Marti et al., 2021). For instance, during the outbreak of COVID-19, some workers at beef plants in Brazil, the U.S., and Australia tested positive for COVID-19^4^. At the same time, Chinese health authorities found the COVID-19 virus on frozen beef samples imported from Brazil, New Zealand, and Bolivia^5^. In the U.S., although the U.S. Centers for Disease Control and Prevention (CDC) and the Food and Drug Administration (FDA) claim that there is no evidence that handling and consuming food causes COVID-19, many consumers are not convinced. They are still concerned about obtaining viruses by consuming food (McFadden et al., 2021). In China, beef and pork imports in November 2020 were almost 70 percent lower than the import volumes in July 2020^6^, and meat prices were also increasing. Although the declining import was partially due to the breakdown of the global supply chain, one of the key reasons was decreasing import demand, as more consumers and restaurants chose domestically produced meat instead of imports.

Although the COVID-19 pandemic seems to be over worldwide, understanding COVID-19-induced stigmas of imported cold-chain food among Chinese consumers and its lasting effect is still critical. China is the world’s largest agricultural importer. Its meat import accounts for 23%, 26%, and 36% of global trade for beef, pork, and lamb, respectively^7^. When China still had a relatively restrictive policy to control COVID-19, the rest of the world had relaxed restrictions (Ding & Zhang, 2022; Emanuel et al., 2022). With the gradually recovered food supply chain, agricultural exporters needed markets for their products by re-accessing the old market or developing new markets. In either situation, the Chinese market is among the most important markets for most agricultural exporters. A better understanding of Chinese consumers’ response to imported cold-chain products when there was a risk of transmitting the virus from food to humans helps estimate the impact of similar viruses on the status of imported cold-chain food stigmas and determine the underlying factors affecting the stigma. This information could provide policymakers and domestic/global agribusiness essential information to develop tailored policies or strategies to better respond to the impact of viruses, such as COVID-19, on food systems.

### 1.2. Literature Review

Much evidence suggests that there may be evolving food stigmas of imported cold-chain food. Stigma is an unwarranted level of avoidance behavior (Walker, 2013), which has been documented for food produced using pesticides, growth hormones, genetically modified organisms, and recycled water (Messer et al., 2017; Scott et al., 2016). Consumers were hesitant about purchasing any item that could be in contact with the virus, including raw and packaged products (Faour-Klingbeil et al., 2021). Faour-Klingbeil et al.’s (2021) study showed that 70% of the respondents were concerned about the risk of transmission of COVID-19 through food. Up to 27% of the respondents were extremely concerned about touching contaminated surfaces during food shopping, and 34% were very concerned about being infected by others during shopping. The situation is also similar to the case of radiation-contaminated food in Japan. Since the Fukushima Daiichi Nuclear Power Plant explosion in March 2011, public anxiety over the radioactive contamination of food and the environment has become widespread (Tajima et al., 2016). The prices of vegetables grown in Fukushima Prefecture decreased by 10–36% after the disaster compared with the counterfactual estimates in the absence of a perceived radiation risk (Tajima et al., 2016). In addition, 70.1% of consumers were less willing to pay for beef from the affected area even when radioactivity was not detected (Hiromi et al., 2012).

This study situates food stigma within the broader context of irrational avoidance behavior, a concept rooted in behavioral economics and psychology. Irrational avoidance occurs when individuals overestimate risks or misinterpret probabilities due to cognitive biases, leading to persistent avoidance despite scientific evidence (Kahneman & Tversky, 1979). This aligns with affect heuristic theory (Slovic et al., 2005), where emotional responses (e.g., disgust and fear) dominate rational risk assessments. For instance, consumers may irrationally avoid imported cold-chain food due to symbolic contamination fears, even when virus transmission risks are negligible.

## 2. Methods and Data

### 2.1. Survey Design

An online survey was developed on COVID-19-induced stigmas of imported cold-chain food among Chinese consumers after the products tested positive for SARS-CoV-2. The online survey was hosted on the Changsha Ranxing Information Technology Ltd. China (Wenjuanxing, www.wjx.cn) platform. The survey was divided into three sections, as follows: 

(i) Demographics and behavior characteristics, such as age, gender, income, and previous purchase frequency of imported cold-chain food.

(ii) Consumers’ food safety risk perception of imported cold-chain food. Specifically, we measured consumers’ risk perception, including the perception of imported cold-chain food contaminated with Novel Coronavirus entering the market and the perception of getting COVID-19 from cold-chain food. To measure consumers’ perception of imported cold-chain food contaminated with Novel Coronavirus entering the market, respondents were asked, “What do you think of the risk of imported cold-chain food contaminated with Novel Coronavirus entering the market?” The perception of getting COVID-19 from cold-chain food was measured by the question, “What do you think is the risk of getting COVID-19 from cold-chain food?”

Furthermore, we used the same statistical procedures to examine consumers’ perceptions of the contamination risk of beef from different countries. Respondents were asked, “What do you think of the risk of imported cold-chain beef contaminated with COVID-19 from the following countries: the U.S., Australia, and Brazil?” The response options for all questions consisted of five-point Likert scales ranging from “extremely high risk” to “extremely low risk”. These three countries were selected because China ranks as the third-largest export market for U.S. beef, and the total value of U.S. beef to China surged 413% from 2020 to 2021^8^. China is the top export market for Australian beef, with a total import value of USD 2.67 billion in 2019^9^. Brazil was the largest beef exporter in the world in 2018, accounting for 20% of the total global beef exports, and China was the top beef export market for Brazil in 2018^10^.

(iii) Consumers’ risk perception of and willingness to pay (WTP) for beef imported from the U.S., Australia, and Brazil. Finally, we used a payment card method to estimate consumers’ WTP for imported cold-chain beef steak. The payment card method was developed by Mitchell and Carson (Mitchell & Carson, 1981) and has been used in many recent studies (Wang et al., 2021; Yu et al., 2014). Previous studies showed that respondents were more likely to answer payment card questions, which can result in more robust mean and median values of WTP (Donaldson et al., 1997; Ready et al., 2001). We firstly asked respondents to assume that the price for a piece of domestic frozen filet mignon (150 g) is 20 yuan^11^, then requested them to pick the maximum acceptable price for the same piece of frozen filet mignon from different countries before COVID-19 and now. The choices of prices we set in the payment card included nine intervals: under 16 yuan, 16–18 yuan, 18–20 yuan, 20–22 yuan, 22–24 yuan, 24–26 yuan, 26–28 yuan, 26–30 yuan, and 30 yuan or above. Detailed information on the survey questions is illustrated in Table 1.

In order to measure the impact of COVID-19-induced stigmas of imported cold-chain food among Chinese consumers over time, seven rounds of nationally representative surveys were conducted every one or two months, involving some standard questions. We conducted the first online survey on 20–28 December 2020, just one year after the outbreak of COVID-19. Since June 2020, several Chinese cities, including Beijing, Dalian, Qingdao, and Tianjin, have reported cases related to cold-chain transportation^12^. On 23 July, China’s State Council issued a guideline demanding all imported meat products must be provided with a nucleic acid test certificate before coming into industrial plants for processing^13^. The following additional surveys were conducted at intervals of about one or two months, on 20–23 January, 19–23 March, 20–23 April, 27–28 May, 19–20 July, and 1–2 October 2021 (Table 2).

### 2.2. Econometric Model

The ordered logit model: An ordered logit model was used to determine the critical factors affecting respondents’ perception of imported cold-chain food contaminated with Novel Coronavirus entering the market (${Perception}_{i}^{1}$) and perception of getting COVID-19 from cold-chain food (${Perception}_{i}^{2}$):

$${Perception}_{i}^{s}=\alpha_{1}{time}_{j}+\alpha_{2}{time}_{j}*{time}_{j}+{\beta X}_{i}+\varepsilon_{i},\;s=1,2\;\&\;j=1,\ldots ,7,$$

where $X_{i}$ represents a vector of demographic variables, such as gender, age, education level, monthly household income, and having children at home, while ${time}_{j}$ is the survey time. The square of the time variable was also added to explore the potential non-linear effect of time on risk perceptions, and$\;\varepsilon_{i}$ is the disturbance term, following a standard logistic distribution.

The interval regression: WTPs from the payment card method consisted of intervals and censoring observations. The interval regression, which is a generalized Tobit model (Amemiya, 1973) for outcomes with interval censoring, specifies the payment card nature of the data (Yang et al., 2014). The differences between censored, truncated, and interval data have been discussed by Cameron and Trivedi (Cameron & Trivedi, 2010). In this study, the known boundaries of WTP, as well as the chosen range, indicate the underlying maximum WTP for imported beef steak.

The empirical specification in this study is shown as follows:

$${WTP}_{i}^{c}=\beta_{0}+\beta_{1}{WTP\_before}_{i}^{c}+\beta_{2}{Perception}_{i}+\beta_{3}{Demo}_{i}+\varepsilon_{i},$$

where the dependent variable (${WTP}_{i}^{c}$) is the chosen category of the payment card question, repressing individual *i*’s willingness to pay for imported beef steak from country *c* (*c* = U.S., Brazil, and Australia). The empirical model in this study consisted of three independent variables: WTP before the COVID-19 pandemic, perception variables (risk perception of contamination with Novel Coronavirus from U.S./Australia/Brazil, perception of getting COVID-19 from cold-chain food, and perception of imported cold-chain food contaminated with Novel Coronavirus entering the market), and demographic and behavioral characteristics (previous purchase frequency of imported cold-chain food, gender, age, education level, income, and children at home). The $\beta_{s}$ are parameters to be estimated, and ε is summed to be a normally distributed error term.

## 3. Results

### 3.1. Sample Description

A professional Chinese data collection company (Wenjuanxing, https://www.wjx.cn) with a 2.6 million sample database was hired to collect data. Rigorous quality control methods were implemented, including (1) pre-survey validation of participants’ demographic information, (2) embedded verification questions requiring respondents to replicate prior answers to detect and exclude inattentive responses, and (3) single-device and single-IP address restrictions. Following the application of these validation procedures, the final analytical sample consisted of 2570 valid participants. This method minimized selection bias by accessing a diverse sample pool (2.6 million users) and allowed real-time quality control checks (e.g., IP validation).

Table 3 gives the explanations for the variables and shows the descriptive statistics. We collected about 300 to 400 samples each time, with a total number of 2570 samples collected from 20 December 2020 to 2 October 2021 (Table 2). Qualified respondents were adults and primary household grocery shoppers. Overall, about 53.7% of the survey respondents were females, which was consistent with the fact that females are the primary food shoppers in China. About 92.5% of the respondents had more than 12 years of education, and the median monthly household income was between 12,001 and 16,000 yuan, representing China’s growing middle-income class, which has the strongest purchasing power in China. In addition, more than 61% of the samples had children at home. Our samples can represent what is generally defined by the National Bureau of Statistics as a family of three earning between 100,000 yuan and 500,000 yuan annually^14^. They are the primary buyers of imported cold-chain food in China.

**Figure 1:**
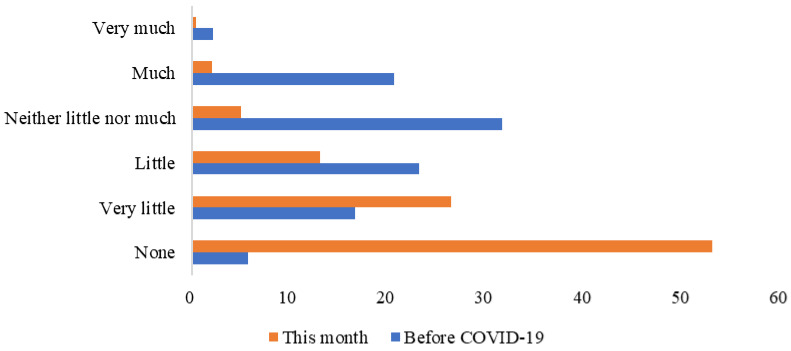
Respondents’ purchase frequency of imported cold-chain food this month (the survey month) and before COVID-19 (%).

Figure 1 reports respondents’ purchase frequency of imported food this month (the survey conducted month) and before COVID-19. Respondents reduced their purchase frequency of imported food in the survey month because they were concerned about transmission of the Novel Coronavirus from imported food. Here, 53.03% of the respondents claimed that they did not purchase imported cold-chain food in the survey month, which was 5.72% before the COVID-19 pandemic. It is clear that consumers’ concerns about the spread of COVID-19 in the food system disrupted the imported food trade market (Lüth et al., 2019), creating potential risks to food security and nutrition, particularly in certain countries (Marti et al., 2021).

### 3.2. Respondents’ Risk Perception of Cold-Chain Beef

**Figure 2:**
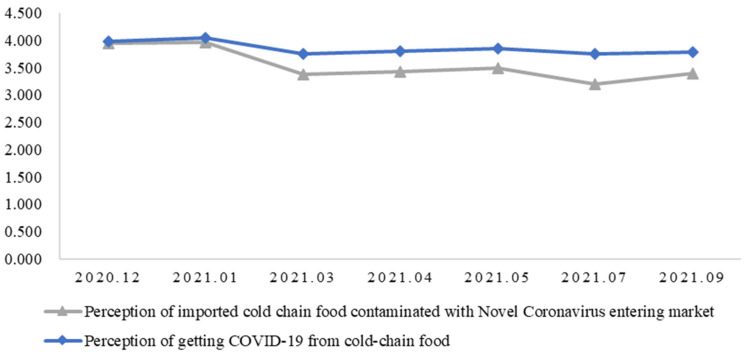
Respondents’ risk perception of imported cold-chain food.

Figure 2 reports respondents’ risk perception of imported cold-chain food. On average, respondents were worried about imported cold-chain food contaminated with Novel Coronavirus entering the market and getting COVID-19 from cold-chain food while purchasing it. As time went by, respondents’ relative degrees of worry about imported cold-chain food contaminated with Novel Coronavirus entering the market fell slightly in March 2021. This may be because China’s Administration for Market Regulation then required imported cold-chain food products to undergo a nucleic acid test for COVID-19 before sale. However, respondents’ perception of getting COVID-19 from cold-chain food almost did not change. This is because although statements from governments suggested that food itself was unlikely to be a carrier of COVID-19, consumers were still concerned about its potential connection with food (McFadden et al., 2021). This is consistent with previous studies that claimed it is difficult to reduce stigmatization (Kecinski et al., 2016; Kecinski & Messer, 2018), and providing information does not necessarily sway wary consumers (McFadden & Lusk, 2015).

**Figure 3:**
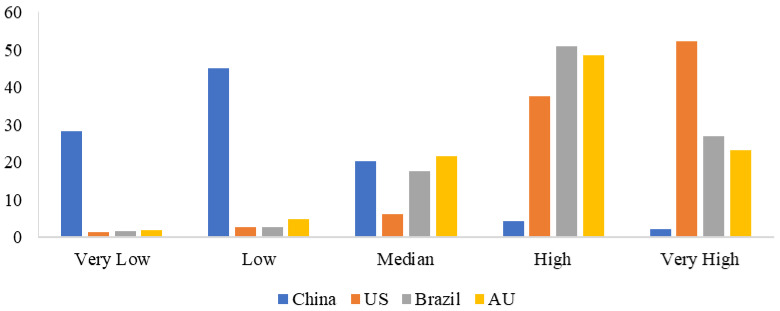
Risk perception of cold-chain beef from different countries contaminated with Novel Coronavirus (%).

**Figure 4:**
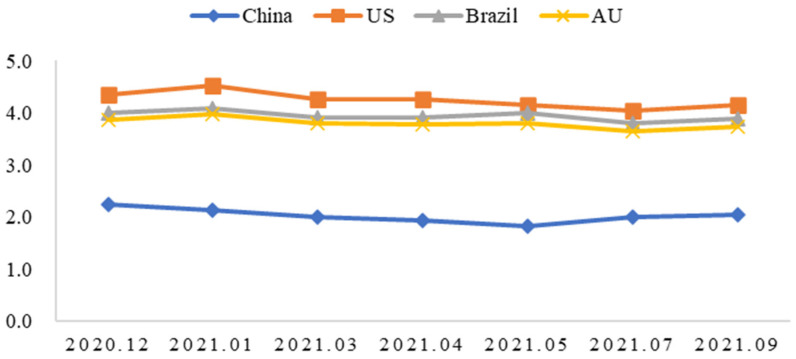
Risk perception of cold-chain beef from different countries contaminated with Novel Coronavirus over time.

Figure 3 reports respondents’ perceived risk of cold-chain beef from different countries contaminated with Novel Coronavirus at the survey time. On average, about 28% and 45% of the respondents believed domestic (Chinese) cold-chain beef carried a very low or low risk of COVID-19, while about 37% and 52% of the respondents thought U.S.-imported cold-chain beef carried a high or very high risk of COVID-19. It was also the same for Australia and Brazil. Furthermore, Figure 4 reports respondents’ perceived risk of cold-chain beef from different countries contaminated with Novel Coronavirus over time. Overall, respondents perceived U.S.-imported cold-chain beef to carry the highest risk of contamination with Novel Coronavirus, followed by those from Brazil and Australia. As time went by, there was only a small fall.

### 3.3. Willingness to Pay for Imported Cold-Chain Beef

**Figure 5:**
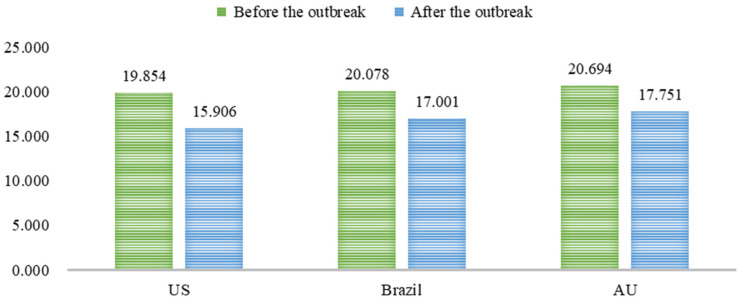
Respondents’ willingness to pay for imported cold-chain beef before and after COVID-19 (in yuan).

**Figure 6:**
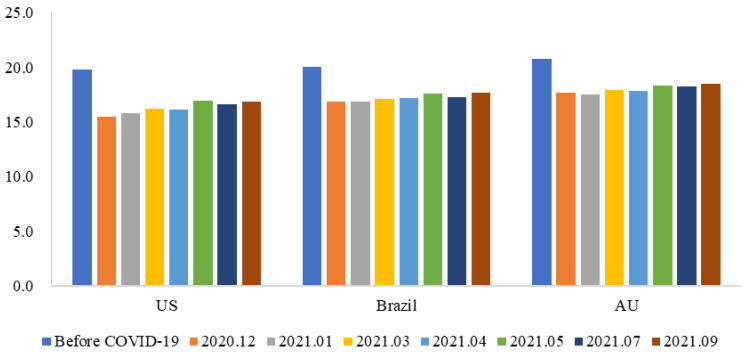
Respondents’ willingness to pay for cold-chain beef from different countries over time (in yuan).

Figure 5 reports respondents’ willingness to pay for imported cold-chain beef from the U.S./Australia/Brazil. Different from a previous study that showed that Beijing consumers were willing to pay more for Australian beef products than for U.S. or domestic (Chinese) beef (Ortega et al., 2016), Chinese consumers were willing to pay more for domestic beef than for imported beef after the information shock of imported cold-chain food testing positive for SARS-CoV-2. Assuming the price of a piece of domestic cold chain filet steak was 20 yuan, before COVID-19, the mean willingness to pay for a piece of cold chain filet steak (150 g) imported from the U.S. was 19.854 yuan, which is less than that for the steak imported from Australia (20.694 yuan) and Brazil (20.078 yuan). This aligns with a previous study by Wang et al. (2024), indicating that political conflicts affecting consumers’ emotions and changing their perception of a country’s image can impact their preferences. Respondents’ willingness to pay for all imported cold-chain beef fell significantly in light of the impact of imported cold-chain products testing positive for SARS-CoV-2. Respondents’ willingness to pay for cold-chain beef from the U.S. fell by about 4 yuan per unit and about 3 yuan per unit for that from Australia and Brazil. Furthermore, Figure 6 shows that as time passed, there was only a slight recovery in respondents’ willingness to pay for imported cold-chain beef. This implies a lasting impact of COVID-19-induced stigmas of imported cold-chain food among Chinese consumers.

### 3.4. Respondents’ Characteristics Associated with Risk Perception of Imported Cold-Chain Beef

Table 4 presents the ordered logit coefficient for respondents’ characteristics associated with risk perception of imported cold-chain food. On average, elderly respondents were more likely to perceive a high risk of imported cold-chain food contaminated with Novel Coronavirus entering the market, and they also perceived a high risk of getting COVID-19 from cold-chain food. This is different from a study of American consumers, which indicated that the elderly were less likely to be concerned about transmission while shopping for food (McFadden et al., 2021). Respondents with children in the family were more likely to perceive a high risk of imported cold-chain food contaminated with Novel Coronavirus entering the market, and they also perceived a high risk of getting COVID-19 from cold-chain food. Most income dummy variables were positive, and only Income5 (16,000 < monthly family income < 20,000) was significant, which is consistent with the fact that those who had a higher income were more likely to be concerned (McFadden et al., 2021). The education level variable was not significant, which is consistent with Hao and Wang (2021)’s study, which showed that simple scientific education is ineffective for stigmatized people. McFadden et al. (2021) also showed that more educated consumers were more likely to be concerned about transmission while shopping for food.

Interestingly, the coefficient of time was negative and significant, while the coefficient of time squared was positive. This indicates that as time went by, respondents’ risk perception of imported cold-chain food was likely to fall, but the speed of falling was likely to be low, which implies that stigmas of imported cold-chain food would lead to persistent and long-term adjustments to consumer food behavior (McFadden et al., 2021). As the COVID-19 pandemic went on, fear-based responses resulted in stigmatizing products, which is hard to eliminate over a long time (Schulze & Wansink, 2012). This is similar to a study in Japan, which showed that Japanese consumers have been sensitive to the risk of radiation exposure via food consumption (Shimokawa et al., 2018). As there is no evidence to show that consuming imported cold-chain food causes COVID-19 and imported cold-chain food products have to undergo a nucleic acid test for COVID-19 before the sale, individuals may understand the associated risk from an objective and scientific perspective and may not be fearful. However, they still become stigmatized because their subjective reasoning and their visceral reaction to contaminated items may be interpreted as disgust (Rozin, 2001).

### 3.5. Factors Influencing Willingness to Pay for Imported Cold-Chain Beef

Interval regression coefficients for respondents’ characteristics and risk perception associated with respondents’ willingness to pay for imported cold-chain beef from the U.S./Australia/Brazil are shown in Table 5. The results showed that respondents who perceived a higher risk of contamination with Novel Coronavirus from the U.S./Australia/Brazil were more likely to pay a discount for imported cold-chain beef. Those who perceived a higher risk of getting COVID-19 from cold-chain food and imported cold-chain food contaminated with Novel Coronavirus entering the market were likely to pay a discount for imported cold-chain beef. This implies that high-risk perception reduced respondents’ willingness to pay for imported cold-chain beef, which is consistent with the fact that food safety perceptions are the key to strongly influencing consumers’ decision-making (Grunert, 2002; Neill & Holcomb, 2019). Food stigma is fueled by a set of multiple subjective risk perceptions, which all affect consumers’ demand for stigmatized food and can lead to large monetary losses even when there are no associated risks (Kecinski et al., 2016).

Furthermore, the absolute coefficient value of risk perception of contamination with Novel Coronavirus from the U.S./Australia/Brazil was larger than the perception of contamination with Novel Coronavirus entering the market and the perception of getting COVID-19 from cold-chain food. This implies that the perception of risk origin had a larger impact on consumer behavior. The elderly respondents were less likely to pay for imported cold-chain beef. Those who often purchased imported cold-chain food were more likely to pay a higher price for imported cold-chain beef. Those willing to pay a high price for imported cold-chain beef were also willing to pay a high price after the COVID-19 pandemic. This implies the power of habit.

## 4. Discussion and Conclusions

The COVID-19 pandemic has affected the food system directly and indirectly. Understanding the stigmatization of imported cold-chain food during the pandemic is vital for stabilizing the global food trade and economic recovery. In this study, we surveyed 2570 Chinese consumers in December 2020 and January, March, April, May, July, and October 2021 to determine the changes in Chinese consumers’ risk perception and willingness to pay for imported cold-chain food over time.

The results suggested COVID-19-induced stigmas of imported cold-chain food among Chinese consumers. Chinese consumers’ concern about getting COVID-19 from cold-chain food has sustained for a long time, even if governments’ statements suggested food was unlikely to be a carrier of COVID-19. The results are consistent with a study (McFadden et al., 2021) about American consumers. Our results also showed that although there was a slight fall in risk perception in March 2021, it did not keep falling over time. In addition, although imported meats had been considered high quality before the COVID-19 pandemic in China, respondents’ willingness to pay for beef steak from the U.S. fell by about 4 yuan, and 3 yuan for those from Australia and Brazil. Furthermore, as time passed, consumers were only slightly more willing to pay for imported cold-chain beef. This is consistent with previous studies that claim it is difficult to reduce stigmatization (Kecinski et al., 2016; Kecinski & Messer, 2018), and providing information does not necessarily sway consumers away from the concerns about the risk of COVID-19 contamination (McFadden & Lusk, 2015). Our findings align with affect heuristic theory (Slovic et al., 2005), where emotional responses to contamination fears dominate rational assessments of scientific safety. This mirrors GMO stigma research, where moral opposition persists despite regulatory approvals (Scott et al., 2016). These results underscore the need for policy interventions, such as blockchain-based traceability systems (Lin et al., 2022), to rebuild trust. For industries, targeted messaging emphasizing safety compliance could mitigate aversion among risk-sensitive groups, as shown in prior stigma mitigation efforts (Kecinski & Messer, 2018).

Our study identified some key factors associated with the risk perception of imported cold-chain food. Elderly respondents or those with children were more likely to perceive a higher risk of imported cold-chain food contaminated with Novel Coronavirus. Interestingly, the risk perception decreased over time but at a slower rate, indicating that consumers’ risk perception of imported cold-chain food was likely to fall as time went by. Still, it would stay at a high level for an extended period. The results of the interval regression showed that a high risk perception reduced respondents’ willingness to pay for imported cold-chain beef. Those who often purchased imported cold-chain food were more likely to pay a high price for imported cold-chain beef. Our results implied that stigmas of imported cold-chain food would generally lead to persistent and long-term consumer behavior adjustments (McFadden et al., 2021). The factors affecting the stigmas varied by consumers depending on several important factors, such as age, having children at home, and risk perceptions.

While the COVID-19 pandemic is almost over, it has directly or indirectly affected the global market and food supply chains. Our results implied that stigma toward imported cold-chain food created significant barriers to food security and economic recovery during the pandemic. Specifically, consumers’ sustained risk perceptions led to reduced demand for imported meats, particularly from the U.S., Australia, and Brazil. This decline in willingness to pay likely disrupted supply chains, as Chinese consumers historically viewed these imports as high-quality and reliable. Such shifts in demand could have destabilized global trade flows, affecting both exporting nations’ economies and China’s access to diverse food sources—key components of food security. In addition, reduced imports may force countries to rely on domestic production or alternative suppliers, potentially increasing prices or lowering quality. Additionally, the persistence of stigma suggests that even after the pandemic, these behavioral adjustments could slow economic recovery, as consumer trust in imported food remains impaired.

While our study focused on Chinese consumers, the implications extend globally. Future research can explore how stigma mitigation strategies—such as transparent communication or traceability systems—could restore consumer confidence and facilitate trade resilience. Understanding these dynamics is critical for policymakers aiming to balance public health messaging with economic stability.

## Figures and Tables

**Table 1:** Survey questions.

Variable	Description	Definition
Previous purchase frequency of imported cold-chain food	How often do you purchase imported cold-chain food?	1 = none, 6 = very much
Perception of imported cold-chain food contaminated with Novel Coronavirus entering the market	What do you think of the risk of imported cold-chain food contaminated with Novel Coronavirus entering the market this month?	1 = extremely low risk, 5 = extremely high risk
Perception of getting COVID-19 from cold-chain food	What do you think is the risk of getting COVID-19 from cold-chain food?	1 = extremely low risk, 5 = extremely high risk
Risk perception of contamination with Novel Coronavirus from the U.S./Australia/Brazil	What do you think of the imported cold-chain food contaminated with Novel Coronavirus from the following countries: U.S./Australia/Brazil?	1 = extremely low risk, 5 = extremely high risk
Willingness to pay for cold-chain beef from different countries	Assume that the price for a piece of domestic frozen filet mignon (150 g) is 20 yuan. What is the maximum acceptable price for the same piece of frozen filet mignon from the U.S./Australia/Brazil?	Under 16 yuan, 16–18 yuan, 18–20 yuan, 20–22 yuan, 22–24 yuan, 24–26 yuan, 26–28 yuan, 26–30 yuan, or 30 yuan or above.

**Table 2:** Survey time.

Survey	Time	Size of Sample
Survey round 1	20–28 December 2020	340
Survey round 2	20–23 January 2021	434
Survey round 3	19–23 March 2021	379
Survey round 4	20–23 April 2021	374
Survey round 5	27–28 May 2021	330
Survey round 6	19–20 July 2021	334
Survey round 7	1–2 October 2021	379

**Table 3:** Descriptive statistics.

Variable	December 2020 (N = 322)	January 2021 (N = 418)	March 2021 (N = 355)	April 2021 (N = 364)	May 2021 (N = 317)	July 2021 (N = 323)	October 2021 (N = 379)	Pooled Sample (N = 2570)
Female	0.579	0.502	0.544	0.543	0.506	0.524	0.565	0.537
Age	30.753	30.917	30.422	30.610	31.485	30.895	30.583	30.798
Education level	0.874	0.924	0.942	0.925	0.936	0.907	0.958	0.925
Income1	0.059	0.035	0.050	0.051	0.036	0.048	0.042	0.046
Income2	0.179	0.184	0.172	0.160	0.130	0.153	0.113	0.157
Income3	0.229	0.244	0.219	0.233	0.209	0.231	0.211	0.226
Income4	0.206	0.182	0.219	0.235	0.252	0.207	0.208	0.214
Income5	0.112	0.196	0.137	0.150	0.188	0.138	0.169	0.157
Income6	0.115	0.078	0.116	0.102	0.121	0.129	0.145	0.114
Income7	0.100	0.081	0.087	0.070	0.064	0.096	0.111	0.087
Children	0.597	0.560	0.657	0.586	0.682	0.665	0.720	0.636

Note: Female (female = 1; male = 0), age (respondents’ age, years), education level (more than 12 years of education = 1; otherwise = 0), income1 (monthly family income < 4000 = 1; otherwise = 0), income2 (4000 < monthly family income < 8000 = 1; otherwise = 0), income3 (8000 < monthly family income < 12,000 = 1; otherwise = 0), income4 (12,000 < monthly family income < 16,000 = 1; otherwise = 0), income5 (16,000 < monthly family income < 20,000 = 1; otherwise = 0), income6 (20,000 < monthly family income < 24,000 = 1; otherwise = 0), income7 (24,000 < monthly personal income = 1; otherwise = 0), and children (having children = 1; otherwise = 0).

**Table 4:** Estimation of the ordered logit models.

	Perception of Imported Cold-Chain Food Contaminated with Novel Coronavirus Entering Market	Perception of Getting COVID-19 from Cold-Chain Food
Survey time	−0.759 ***	−0.279 ***
	(0.093)	(0.093)
Survey time squared	0.0636 ***	0.0227 **
	(0.011)	(0.011)
Female	0.074	−0.0586
	(0.076)	(0.078)
Age	0.0147 ***	0.0110 **
	(0.005)	(0.005)
Education level	0.140	−0.0976
	(0.154)	(0.159)
Income2	0.106	0.0246
	(0.206)	(0.211)
Income3	0.148	0.0595
	(0.202)	(0.207)
Income4	0.259	0.109
	(0.204)	(0.209)
Income5	0.417 **	0.233
	(0.211)	(0.217)
Income6	−0.0151	−0.0147
	(0.219)	(0.225)
Income7	0.192	0.273
	(0.229)	(0.236)
Children	0.242 ***	0.324 ***
	(0.083)	(0.085)

Note: ** Significant at the 5% level. *** Significant at the 1% level.

**Table 5:** Estimation of the interval regression models.

Variables	Imported U.S.	Imported Brazil	Imported Australia
WTP_before_	0.576 ***	0.535 ***	0.527 ***
	(0.019)	(0.017)	(0.017)
Risk perception of contamination with Novel Coronavirus from U.S./Australia/Brazil	−1.288 ***	−0.851 ***	−0.884 ***
	(0.130)	(0.103)	(0.104)
Perception of getting COVID-19 from cold-chain food	−0.586 ***	−0.322 ***	−0.423 ***
	−0.139	−0.109	−0.114
Perception of imported cold-chain food contaminated with Novel Coronavirus entering the market	−0.171	−0.189 **	−0.219 **
	(0.122)	(0.096)	(0.100)
Previous purchase frequency of imported cold-chain food	0.320 ***	0.221 ***	0.307 ***
	(0.094)	(0.074)	(0.077)
Female	−0.0799	0.0891	0.0454
	(0.215)	(0.170)	(0.176)
Age	−0.0353 **	−0.0158	−0.0273 **
	(0.015)	(0.012)	(0.012)
Education level	−0.417	−0.205	−0.513
	(0.451)	(0.354)	(0.366)
Income2	−0.105	−0.235	−0.244
	(0.608)	(0.469)	(0.495)
Income3	0.331	−0.0327	0.1
	(0.593)	(0.458)	(0.482)
Income4	−0.551	−0.533	−0.182
	(0.600)	(0.464)	(0.488)
Income5	−0.266	−0.00448	−0.00736
	(0.619)	(0.478)	(0.503)
Income6	−0.556	−0.759	−0.515
	(0.643)	(0.499)	(0.524)
Income7	−0.736	−0.732	−0.553
	(0.670)	(0.521)	(0.546)
Children	0.349	0.0576	0.0094
	(0.238)	(0.187)	(0.195)

Note: ** Significant at the 5% level. *** Significant at the 1% level.

## Data Availability

The original contributions presented in this study are included in the article. Further inquiries can be directed to the corresponding author(s).

## Notes

1 UPDATED: Timeline of the Coronavirus|Think Global Health, n.d. (https://www.thinkglobalhealth.org/article/updated-timeline-coronavirus (accessed on 12 July 2022)).

2 State-by-State Coronavirus-Related Restrictions. AARP (https://www.aarp.org/politics-society/government-elections/info-2020/coronavirus-state-restrictions.html (accessed 12 July 2022)).

3 UPDATED: Timeline of the Coronavirus|Think Global Health, n.d. (https://www.thinkglobalhealth.org/article/updated-timeline-coronavirus (accessed on 12 July 2022)).

4 Brazil’s Marfrig says 25 workers tested positive for COVID-19 at beef plant (https://www.reuters.com/article/health-coronavirus-marfrig-idUSL1N2D70EJ/ (accessed on 12 July 2022)). Alberta beef plant halts slaughter due to positive COVID-19 test (https://nationalpost.com/pmn/health-pmn/alberta-beef-plant-halts-slaughter-due-to-positive-covid-19-test (accessed 12 July 2022)).

5 COVID-19 virus found on imported frozen beef samples (https://www.thehealthsite.com/news/covid-19-virus-found-on-imported-frozen-beef-samples-780594/ (accessed 12 July 2022)).

6 Chinese consumers are turning away from imported meat due to COVID-19 fears (https://www.thepigsite.com/news/2021/01/chinese-consumers-are-turning-away-from-imported-meat-due-to-covid-19-fears (accessed 12 July 2022)).

7 China accounts for 25% of the global meat trade—UBI Meat Experts in QA, n.d. (https://ubibeefinspection.com/2019/09/03/china-accounts-for-25-of-the-global-meat-trade/ (accessed 12 August 2022)).

8 Beef and Beef Products 2021 Export Highlights (WWW Document), n.d. USDA Foreign Agricultural Service (https://www.fas.usda.gov/beef-2021-export-highlights (accessed 12 August 2022)).

9 Australia’s beef trade with China|Meat and Livestock Australia (WWW Document), n.d. MLA Corporate (https://www.mla.com.au/news-and-events/industry-news/australias-beef-trade-with-china/ (accessed 12 August 2022)).

10 USDA ERS—Brazil Once Again Becomes the World’s Largest Beef Exporter (WWW Document), n.d. (https://www.ers.usda.gov/amber-waves/2019/july/brazil-once-again-becomes-the-world-s-largest-beef-exporter/ (accessed 12 August 2022)).

11 Although the price of a beef steak in China ranged from 12 yuan to more than 100 yuan because of different quality, the dominant price is about 20 yuan, and the payment card includes the most popular prices in the market.

12 China steps up measures in cold-chain transportation to contain winter resurgence of COVID-19, CGTN (https://news.cgtn.com/news/2020-11-13/China-targets-cold-chain-transport-to-contain-winter-spike-of-COVID-19-Vo3p84JDwc/index.html (accessed 12 July 2022)).

13 COVID-19 certificates needed for imported meat, aquatic products (https://www.newsgd.com/node_99363c4f3b/dbd035af80.shtml (accessed 12 July 2022)).

14 Steer the middle course, China Daily (http://www.chinadaily.com.cn/a/202112/28/WS61ca7de3a310cdd39bc7de06.html (accessed 12 July 2022)).
